# Dynamic Responses of Sandwich Beams with Polymethacrylimide (PMI) Foam Cores When Subjected to Impact Loading

**DOI:** 10.3390/ma16031108

**Published:** 2023-01-27

**Authors:** Mousab Mahgoub, Yongqiang Zhang, Chen Yang, Zhuhua Tan

**Affiliations:** 1 School of Mechanical Engineering, Hebei University of Technology, Tianjin 300401, China; 2 Institute of Fluid Physics, China Academy of Engineering Physics, Mianyang 621900, China

**Keywords:** sandwich structure, low-velocity impact, failure mode, energy absorption, impact force

## Abstract

This paper focusses on the load-sustaining and transfer mechanisms of sandwich beams with various types of PMI foam cores under low-velocity impact loading. In the case of quasi-static loading, the different failure modes, failure loads, and deflections were obtained, which agreed well with the results predicted by the theory of sandwich structure. In the case of impact loading, the clamped sandwich beams were subjected to the impact of a striker bar with a momentum of 10 kg∙m/s to 20 kg∙m/s. The de-acceleration of the strike bar was measured to analyze the impact force and energy absorption, and the corresponding failure modes were also obtained. The results showed that the impact force and the corresponding duration time increases with the increases in the thickness of the face sheet and the density of the core. In addition, the failure modes of the sandwich beams transferred from the shear failure mode to the tensile failure mode, which was attributed to the strength ratio between the bottom face sheet and the core. In combination with the experimental results and the plastic hinge theory, the deformation mechanisms of the different sandwich beams are also discussed.

## 1. Introduction

Sandwich structures consists of two face sheets, a core, and an adhesive layer, and they are widely used in aerospace, marine, wind energy, satellite launch vehicle, and civil engineering applications due to the advantages they bring via their mechanical and physical properties [1,2,3,4,5,6,7,8,9,10]. It is clear that most of the suffered load is absorbed by the core of the sandwich structure, which is a vital role in the design and manufacturing of sandwich structures. Much work has been performed on sandwich structures with different types of cores [11,12,13,14,15], for instance, polymeric foam [11], truss [12], metallic foam [13], lattice core [14], and honeycomb [15] types have been used. Still, it remains a challenge for the researchers to develop and design a core with high force-resistance and energy absorption capabilities.

Recently, PMI foam materials with closed cells have attracted considerable interest due to their having a high specific strength, aa high specific stiffness, and excellent energy absorption [16,17,18,19,20,21]. Additionally, there is an increasing demand for lightweight PMI foam and corresponding sandwich structures in specific applications, such as in aircraft and ships, which involve a complicated load case in a service environment. In this context, it is necessary to develop a thorough understanding of the mechanical and physical properties of PMI foam and the related sandwich structures. Much research has been conducted on PMI foam to evaluate the mechanical properties and energy absorption of PMI foam materials, including their macroscopic, quasi-static mechanical properties [16], the influence of the microstructure on a geometric scale [17], the effects of the strain rate and temperature [18,19,20], and constitutive equations [21]. The above-cited research studies paved the way for the application of PMI foam in sandwich structures.

At present, PMI foams are widely used in sandwich structures, which were designed to be used as components in helicopter, radar antenna, fire resistance technologies, etc. [22,23,24], and the mechanical performance of sandwich structures with PMI foam cores have also been investigated experimentally and numerically [25,26,27,28,29,30,31,32,33,34,35,36]. However, most of these studies focused on the case of quasi-static loads, including indentation [25,26], three-point bending [27,28,29], energy absorption [30,31,32,33], and damage and fatigue [34,35,36]. Compared with research on sandwich structures with PMI foam cores under quasi-static loads, studies on the dynamic response of PMI foam sandwich structures are few [37,38,39,40,41]. Yang et al. [38] studied the geometric scale effect on the dynamic response of sandwich panels with PMI foam cores. Zhu et al. [39] developed an energy-based analytical model to predict the dynamic response of multilayer sandwich panels, which agreed well with their experimental results.

The above-cited work showed that the dynamic response of a sandwich structure with a PMI foam core was different from the results in the case of a quasi-static load. In addition, the dynamic load sustaining and transfer mechanism of a sandwich structure with a PMI foam core still need to be clarified. Thus, the work on the dynamic deformation mechanism of PMI foam sandwich structures should receive more attention. In the present paper, the dynamic response of sandwich beams with PMI foam cores were investigated under both quasi-static and impact loads. In order to analyze the influence of the thickness of the face sheet, the density of the core, and the thickness of the core on the load sustaining and transfer mechanisms of a sandwich beam, different sandwich beams with various PMI cores were designed. Each sandwich beam was clamped and impacted by a striker bar at different velocities, and the impact force was measured by an accelerometer. Quasi-static, three-point bending experiments were also performed, and the results were compared with those of the impact experiments. The effects of the density and the thickness of the core and the thickness of the face sheet on the failure mode and impact force were analyzed. Combined with the experimental results and plastic hinge theory, the dynamic deformation and failure mechanisms of the sandwich beams with different PMI cores are also discussed.

## 2. Experimental Procedures

### 2.1. Materials and Specimens

PMI foam materials with varying densities and thicknesses were used as the cores of the sandwich beams studied in this paper, and they were supplied by the Suzhou Zhong Bao composite material company, Suzhou, China. Aluminum face sheets alloy (AL-1050) were produced by Lutai Company, China. Two aluminum face sheets were adhered to the surfaces of a PMI foam core using epoxy resin (produced by 3M Scotch-Weld Company, St. Paul, MN, USA). The densities of the PMI foam materials were 52 kg/m^3^, 110 kg/m^3^, and 200 kg/m^3^, and they were coded with the letters A, B, and C, respectively. The dimensions of the sandwich beams were 260 mm in length and 40 mm in width. The face sheets of the sandwich beams were 0.5 mm and 1.0 mm in thickness, and the thicknesses of the cores were 10 mm and 20 mm, respectively. Subsequently, a sandwich beam marked as “A:0.5/10/0.5” included a core density of 52 kg/m^3^, a face sheet thickness of 0.5; and a core thickness of 10. More details are listed in Table 1.

### 2.2. Experimental Apparatus

**Figure 1:**
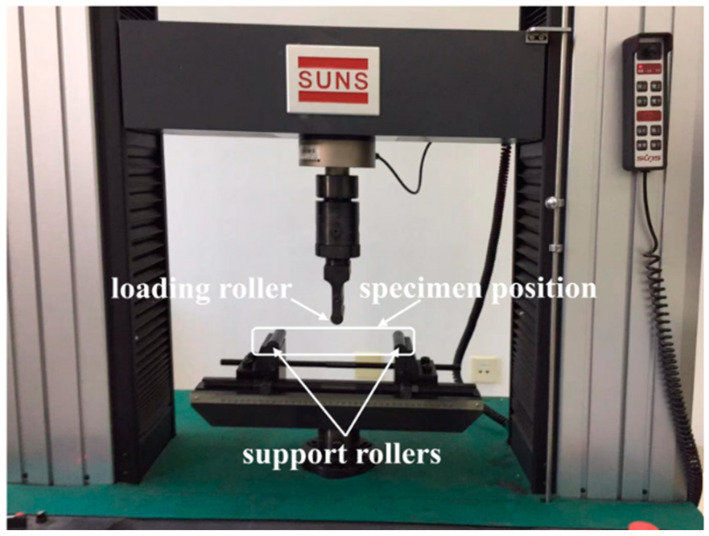
Experimental apparatus for the three-point bending test.

For the case of quasi-static load, three-point bending tests were conducted using a universal test machine, SUNS WAW000 (Shenzhen SUNS Technology Stock Co. Ltd., Shenzhen, China), as shown in Figure 1. Each end of the sandwich beam was supported by two rollers, and the effective length was 200 mm between the two support rollers. The load was applied to the mid-span of the sandwich beam via a steel roller, and the diameter of each of the three rollers was 16 mm. The maximum applied load was 100 kN, and the load rate was controlled by the displacement of the roller at a rate of 2 mm/min [29]. The machine was connected to a computer so as to record the data and calculate the load–displacement curve.

**Figure 2:**
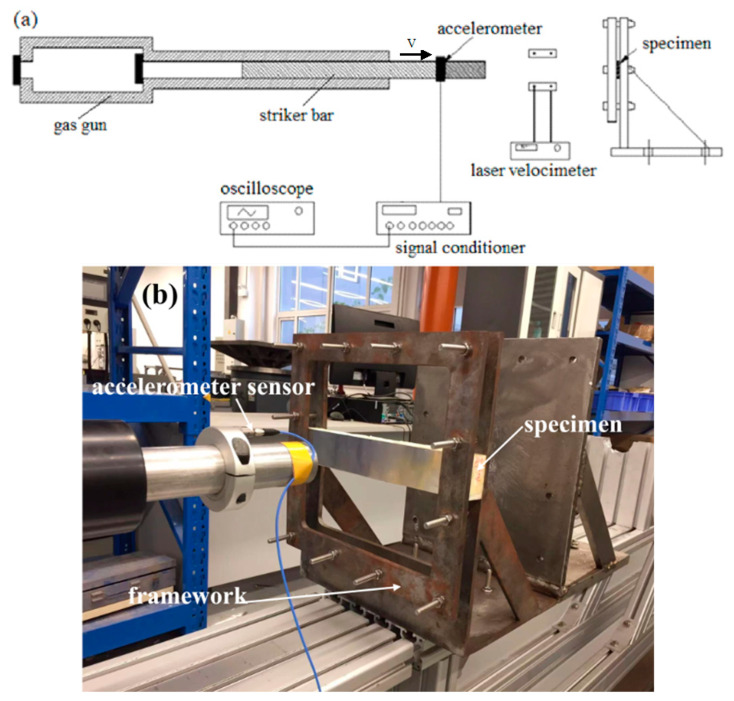
The impact apparatus for the dynamic experiments: (**a**) schematic graph; (**b**) experimental apparatus.

For the impact tests, a revised split-Hopkinson pressure bar was used to perform the impact tests, as shown in Figure 2. A steel striker bar was launched by a gas gun to strike the clamped sandwich beam in its mid-span. The mass of the striker bar was 8 kg, and the dimensions of the striker bar were: 40 mm in diameter and 800 mm in length. The sandwich beam was clamped on a fixed frame, and the effective span of the sandwich beam was 200 mm. The impact velocity of the striker bar was measured by a laser velocimeter. Additionally, a 352C04 PCB accelerometer sensor (PCB company, Depew, NY, USA) was fixed to the striker bar, and it was used to measure the de-acceleration of the striker bar, as shown in Figure 2. The signal of the accelerometer was amplified by the signal conditioner of a PCB 482C05 (PCB company, Depew, NY, USA), and then the signals were recorded using a Tektronix MDO3034 oscilloscope (Tektronix, Beaverton, OR, USA). The data-sampling rate was 5 MHz. The sensitivity of the accelerometer was 9.89 mv/g.

## 3. Results and Discussion

### 3.1. Quai-Static Load Cases

#### 3.1.1. Load–Displacement Curves

**Figure 3:**
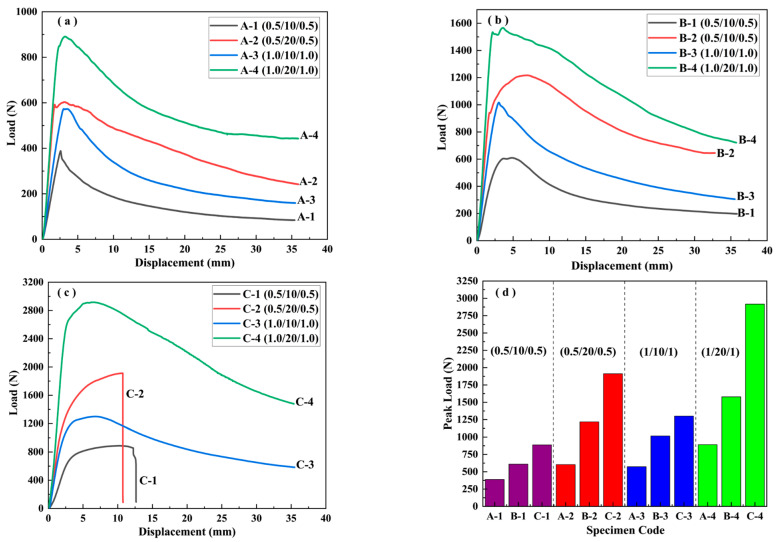
The load–displacement curves of sandwich beams with different core densities under three-point bending: (**a**) A—52 kg/m^3^; (**b**) B—110 kg/m^3^; (**c**) C—200 kg/m^3^; (**d**) the distribution of the peak load.

Figure 3 illustrates the load–displacement curves of the different sandwich beams under the quasi-static, three-point bending loads. As can be seen from Figure 3a–c, the maximum peak load increased from 900 N to 2900 N with the increasing density of the core from 52 kg/m^3^ to 200 kg/m^3^. Generally, the peak load increased with the increase in the density of the PMI core. Moreover, the thicknesses of the core and the face sheet also had an obvious influence on the failure load. In addition, the contribution of the thickness of the core to the peak load is much greater than that of the face sheet. For instance, it is clear from Figure 3a that the peak loads of A-0.5/10/0.5 and A-1/10/1 are about 400 N and 580 N, which is due to the increase in thickness of the face sheet from 0.5 mm to 1.0 mm, respectively. However, the corresponding peak load of A-0.5/20/0.5 reached about 600 N as the thickness of the core increased to 20 mm. A similar tendency also occurred for the density of the core, from 110 kg/m^3^ to 200 kg/m^3^, as shown in Figure 3b,c.

It can be concluded that the load–displacement curves in Figure 3a,b include three stages: an elastic stage, a peak load stage, and a failure stage, which corresponds to the typical indentation failure mode. Because the strength of the foam core was less than that of face sheet, a local indentation failure occurred in the PMI core. Similar results have been reported in Ref. [25]. However, the tendency of the curves for C-0.5/10/0.5 and C-0.5/20/0.5, shown in Figure 3c, is different from that of the other curves, as there is an elastic stage, a hardening stage, and a failure stage, respectively. This means that the strength of the core was greater than that of the face sheet, and the hardening stage corresponded to the tensile deformation process of the bottom face sheet.

#### 3.1.2. Energy Absorption under the Three-Point Bending Load

The energy absorption (*EA*) of the sandwich beam under three-point bending loading can be calculated by the load–displacement curve, and the specific energy absorption is defined by dividing the energy absorption per unit of mass of the beam. The corresponding equations can be expressed as follow [5]:

(1) EA=∫Pdδ

(2) $$SEA=\frac{\int Pd\delta }{M}$$

where P and δ are the corresponding load and displacement in Figure 3, EA is the energy absorption, SEA is the specific energy absorption, and M is the mass.

Moreover, the crush force efficiency, as defined in Ref. [5], is used to analyze the ability of sandwich beams to absorb energy, and the detailed expression is as follows:

(3) $$CFE=\frac{M\cdot L}{P\cdot L}$$

(4) $$M\cdot L=\frac{\int Pd\delta }{\delta }$$

where M·L and P·L are the mean load and peak load, respectively, and CFE is the crush force efficiency.

**Figure 4:**
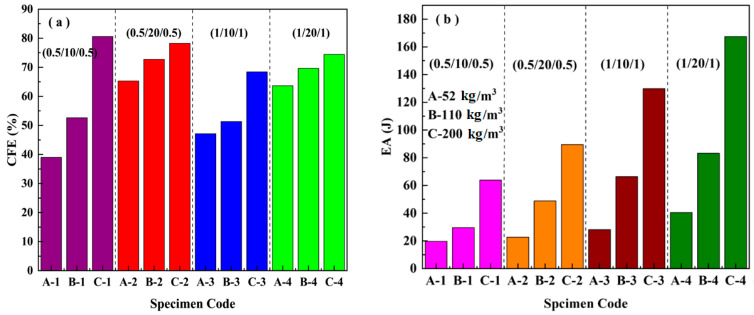

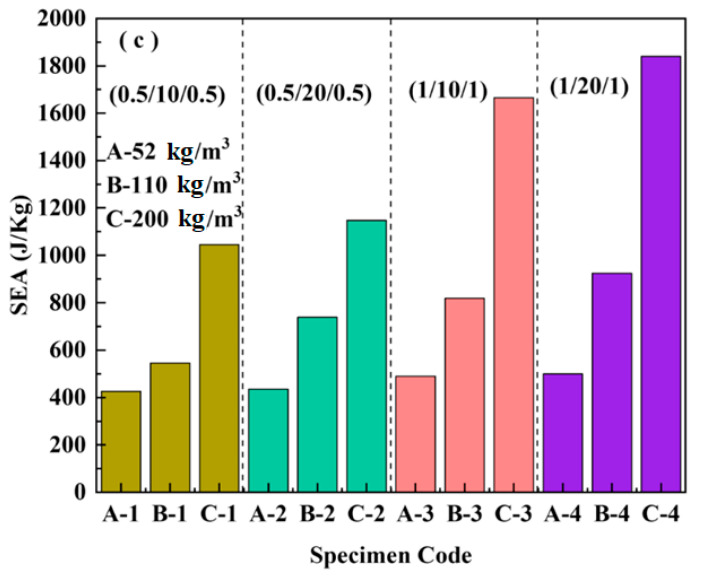
Effects of the density and thickness of the core and face sheet on: (**a**) crush force efficiency; (**b**) energy absorption; (**c**) specific energy absorption.

Figure 4 presents the effects of the density of the core, and the thicknesses of the core and the face sheet on the crush force efficiency, energy absorption, and specific energy absorption. For the crush force efficiency, it is clear from Figure 4a that the density and core thickness had an obvious influence on the CFE; however, the effect of the thickness of the face sheet on the CFE was small. With a core thickness of 10 mm, the CFE increased with the increasing density of the core. However, the increasing tendency decreased when the thickness of the core increased from 10 mm to 20 mm. In regard to energy absorption, it can be observed from Figure 4b,c that the core density and the thickness of the face sheet had significant influences, which is different from the effect on the CFE. Generally, the energy absorption increased with the increase of the thickness of the face sheet, the thickness of the core, and the core density. Moreover, for a specific core density, the contribution to the EA from the thickness of the face sheet was larger than that from the thickness of the core. For example, the EA of C-3 (1/10/1) is larger than that of C-2 (0.5/20/0.5), as shown in Figure 4b.

### 3.2. Dynamic Load Case

#### 3.2.1. Impact Force–Time Curves

**Figure 5:**
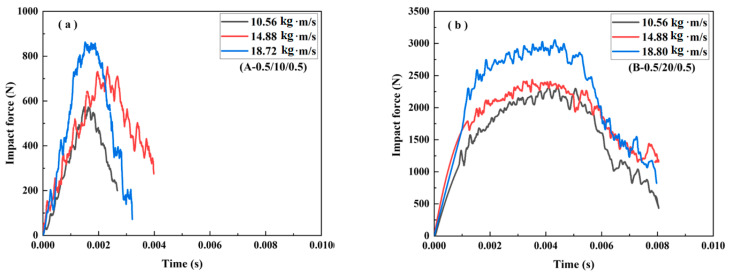

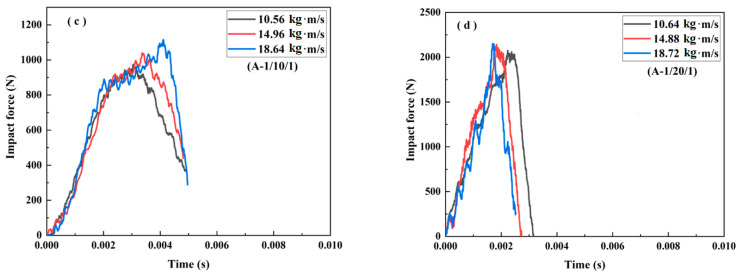
The impact force–time curves of the sandwich beams: (**a**) A-(0.5/10/0.5); (**b**) A-(0.5/20/0.5); (**c**) A-(1.0/10/1.0); (**d**) A-(1.0/20/1.0).

**Figure 6:**
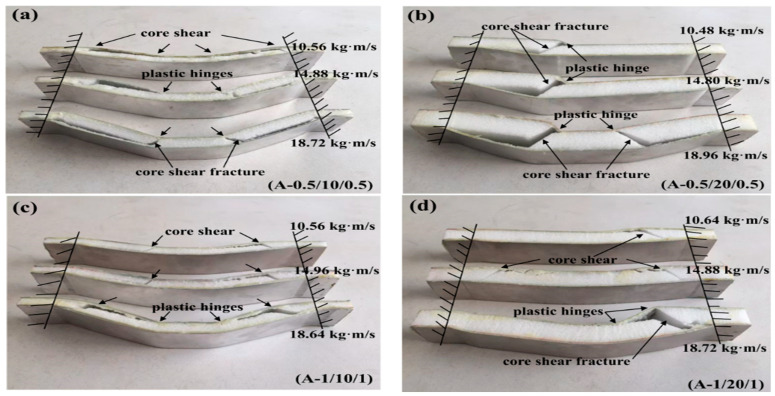
The failure modes of the sandwich beams: (**a**) A-0.5/10/0.5; (**b**) A-0.5/20/0.5; (**c**) A-1.0/10/1.0; (**d**) A-1.0/20/1.0.

**Figure 7:**
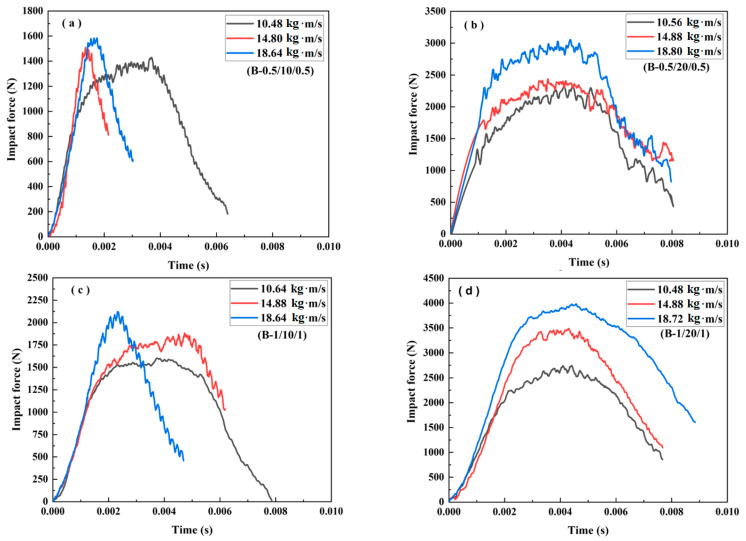
The impact force–time curve of the sandwich beams: (**a**) B-(0.5/10/0.5); (**b**) B-(0.5/20/0.5); (**c**) B-(1.0/10/1.0); (**d**) B-(1.0/20/1.0).

**Figure 8:**
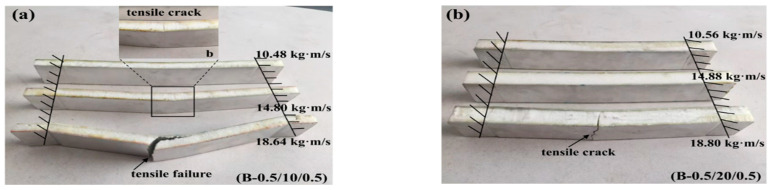

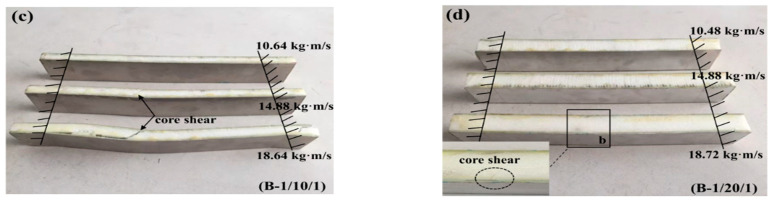
The failure modes of the sandwich beams: (**a**) B-0.5/10/0.5; (**b**) B-0.5/20/0.5; (**c**) B-1.0/10/1.0; (**d**) B-1.0/20/1.0.

**Figure 9:**
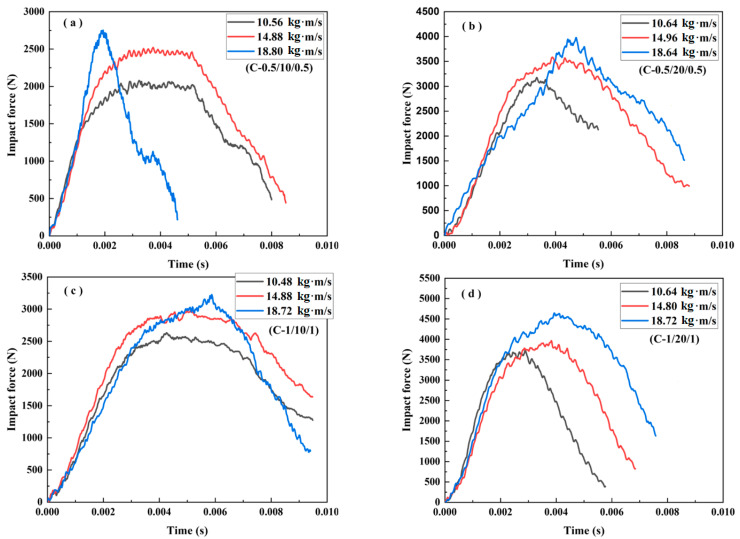
The impact force–time curve of the sandwich beams: (**a**) C-0.5/10/0.5; (**b**) C-0.5/20/0.5; (**c**) C-1.0/10/1.0; (**d**) C-1.0/20/1.0.

**Figure 10:**
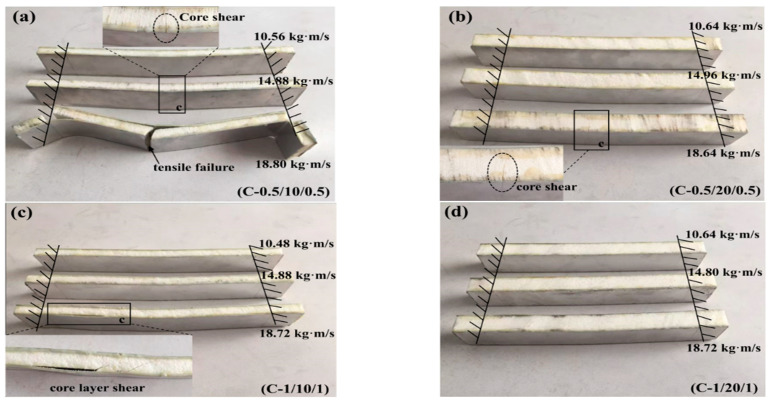
The failure modes of the sandwich beams: (**a**) C-0.5/10/0.5; (**b**) C-0.5/20/0.5; (**c**) C-1.0/10/1.0; (**d**) C-1.0/20/1.0.

The impact force–time curves for the different sandwich beams under various impact velocities were obtained and are shown in Figure 5, Figure 6, Figure 7, Figure 8, Figure 9 and Figure 10, including three load cases. Cases 1–3 correspond to the sandwich beams with core densities of 52 kg/m^3^, 110 kg/m^3^, and 200 kg/m^3^, respectively. The details of the experimental results are as follow:

**Case 1: Core density of 52 kg/m^3^**

Figure 5 and Figure 6 illustrate the impact force–time curves and the corresponding failure modes of the different sandwich beams under different impact velocities, respectively. The impact velocities of the striker bar were 1.32 m/s, 1.86 m/s, and 2.34 m/s, and the corresponding momentum (mv) was 10.56 kg∙m/s, 14.88 kg∙m/s, and 18.72 kg∙m/s, respectively. Generally, the peak of the impact force increased with the increasing impact velocities for each type of specimen, and for the same face sheet, as shown in Figure 5a,b, the impact force increased with the increases in the thickness of the core. The peak impact force for specimen (A-0.5/20/0.5) was 2 times that of specimen (A-0.5/10/0.5), and a similar relationship was also observed for the specimens with face sheets of 1 mm in thickness: (A-1/20/1) and (A-1/10/1).

Moreover, for specimens with the same core thickness, the peak force also increased with the increase in the thickness of the face sheet. For instance, the peak impact force for specimen (A-1/10/1) was 1.25 times greater than that of specimen (A-0.5/10/0.5), and a similar relationship was also found for the specimens with cores that were 20 mm in thickness: (A-1/20/1) and (A-0.5/20/0.5). So, for the type A specimens, the core thickness can enhance suffered, as compared to the thickness of the face sheet.

All of the sandwich beams failed in the core shear failure mode, and there was also a serious deformation present in both the top and bottom face sheets, as shown in Figure 6. The compressive and shear strengths of the PMI core of 52 kg/m^3^ were 1.0 and 0.92 MPa, respectively, which is much less than that of the aluminum face sheet. So, the PMI foam core would fail first when a sandwich beam was subjected to the impact load. It is well-known that there are compressive and tensile stresses in the top and bottom face sheets, respectively, and the PMI core suffered from shear stress. Subsequently, the shear failure occurred in the PMI core, as shown in Figure 6. The energy was also dissipated through the failure of the core and the deformation of the face sheets.

**Case 2: Core density of 110 kg/m^3^**

Figure 7 and Figure 8 illustrate the impact force–time curves and the failure modes of the sandwich beams with a core density of 110 kg/m^3^. It can be noticed that the tendency of the curves in Figure 7 is different from those in Figure 5. Moreover, the failure modes of the sandwich beams in Case 2 (Figure 8) are also significantly different from those of Case 1 (Figure 6). With the exception of the core shear failure mode shown in Figure 8c, all of the sandwich beams almost failed in the bottom face sheet tension failure mode.

It can be seen that the peak impact forces in Case 2 were much larger than those in Case 1, as shown in Figure 5 and Figure 7. Due to the increase in PMI core density of 110 kg/m^3^ in Case 2, the stiffness and strength of the sandwich beams were improved, which also results in a larger bending-resistance. When the striker bar impacted the top face sheet, a bending deformation occurred due to the enhanced bending-resistance. So, the load was sustained in a tensile form by the bottom face sheet, which improved the impact force efficiently. For example, while the face sheet thickness of B-1/10/1 was larger than that of B-0.5/10/0.5, the difference in impact force between these two sandwich beams was small, as shown in Figure 7.

Moreover, the duration time shown in Figure 7 (about 8 ms) was also nearly 2 times that shown in Figure 5 (about 4 ms). This means that the deformation process of the sandwich beam took more time. The duration time was determined by whether the sandwich beam failed, or not. For instance, a serious failure of the sandwich beam can be observed in Figure 8a,c, which corresponds to the short duration time results shown in Figure 7a,c, respectively. A similar tendency in the duration time can also be seen in Figure 5 and Figure 6 for Case 1.

The sandwich beam with a core of 10 mm in thickness failed in both the tensile mode and the shear mode, as shown in Figure 8a,c. However, only the tensile failure mode occurred in the sandwich beam with a core of 20 mm in thickness. Together, the density of 110 kg/m^3^ and the thickness of 20 mm increased the strength and stiffness of the sandwich beam, which sustained the impact of the striker bar. Thus, the impact force and duration time increased with the increase in core density of 110 kg/m^3^, which also improved the energy absorption of the sandwich beam.

**Case 3: Core density of 200 kg/m^3^**

The impact force–time curves of the sandwich beams in Case 3 are shown in Figure 9. The tendency of the curves, as shown in Figure 9, is similar to that shown in Figure 7, and the duration time was also nearly equal, as illustrated by Figure 7. However, the peak impact force values were much larger than those in both Case 1 and Case 2, a trend that we attributed to the increase in the density of the core from 110 kg/m^3^ to 200 kg/m^3^.

It can be observed that there was only one curve with a short duration time, as shown in Figure 9a, which corresponds to the bottom face sheet tensile failure of the sandwich beam shown in Figure 10a. Moreover, there was no obvious failure that occurred in the sandwich beam with a 1 mm thick face sheet and a 20 mm thick core, as shown in Figure 10d. The PMI core density of the 200 kg/m^3^ specimen had greater stiffness and strength than did the 52 kg/m^3^ and 110 kg/m^3^ specimens, and the sandwich beam could sustain much larger load.

As mentioned above, there were two kinds of failure modes for the PMI foam sandwich beam subjected to impact loading, including core shear failure mode and bottom face sheet tensile failure mode. Additionally, the core shear failure mode was caused by the greater strength of the bottom face sheet; however, the greater stiffness of the sandwich beam resulted in the bottom face sheet tensile failure mode.

#### 3.2.2. Energy Absorption under Impact Loading

The energy absorption of the sandwich beam under impact loading can be calculated by the signal of accelerometer. The change of the velocity of the cylindrical projectile ΔV(T) can be expressed by Equation (5) as follows:

(5) $$\Delta V(T)=\int_{0}^{t}a_{0}(t)d(t)$$

Then, the residual velocity of the projectile was as follows:

(6) $$V(t)=V_{0}-\int_{0}^{t}a_{0}(t)d(t)$$

Thus, the displacement of the projectile during the impact process can be obtained:

(7) $$U\left(t\right)=\int_{0}^{t}V\left(t\right)d\left(t\right)$$

Additionally, the impact force can be calculated as follows:

(8) $$P\left(t\right)=ma\left(t\right)$$

where *a*(*t*) is the acceleration, Δ*V*(*t*) and *V*_0_ are the change of the velocity and the initial velocity of the projectile, *P*(*t*) is the impact force, and *U*(*t*) is the displacement. After obtaining the above parameters in Formulas (5)–(8), the load–displacement curve from the formulas of the above-mentioned equations can use Equations (1)–(4)—as mentioned earlier—to calculate energy absorption parameters.

**Figure 11:**
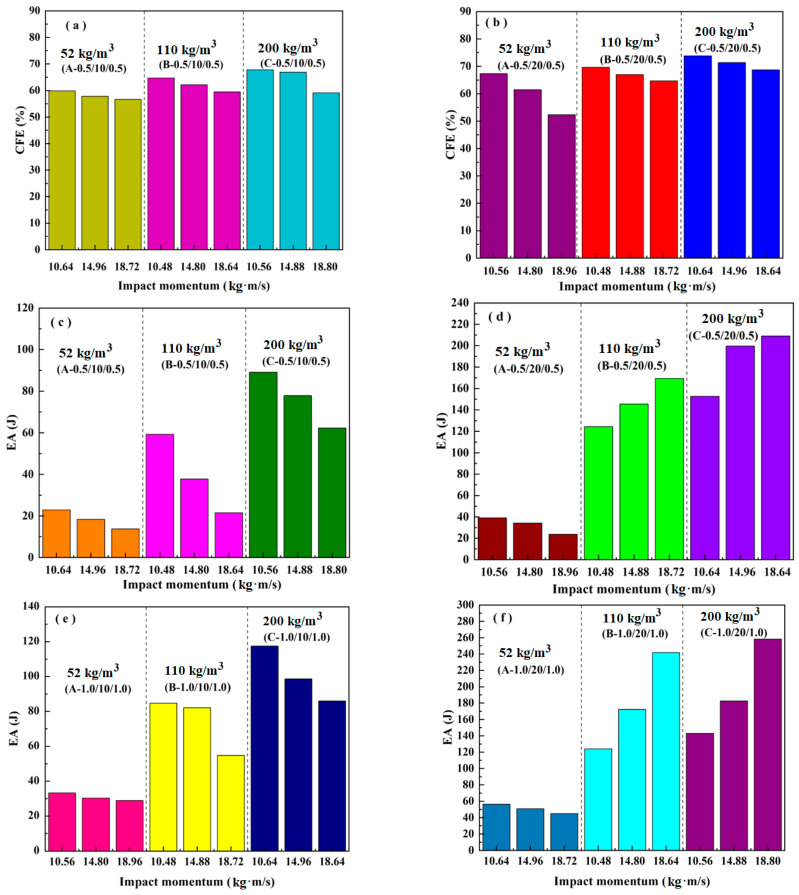
Effects of the impact momentum, density, and thicknesses of the core and face sheet on the crush force efficiency of: (**a**) (0.5/10/0.5); (**b**) (0.5/20/0.5); and energy absorption of: (**c**) (0.5/10/0.5); (**d**) (0.5/20/0.5); (**e**) (1.0/10/1.0); (**f**) (1.0/20/1.0).

Figure 11 presents the effect of the density and thickness of the core and face sheet on the crush force efficiency and energy absorption of the sandwich beam. Generally, the mean impact force is about 60–70% of the peak load for all of the sandwich beams, as shown in Figure 11a,b, which was influenced slightly by the factors of the face sheet and the core. We considered that the duration of the impact force was short, and the load transfer from the face sheet to the core occurred quickly in the sandwich beam. However, the duration of the load transfer in the quasi-static load case was much greater. Thus, the CFE of the impact load case was different from that of quasi-static load case, as shown in Figure 4.

However, the energy absorption was significantly influenced by the density of the core. The energy absorption of the sandwich beams with densities of 110 kg/m^3^ and 200 kg/m^3^ was nearly 2–3 times that of the beam with a density of 52 kg/m^3^. The strength and stiffness of the 52 kg/m^3^ core was much lower than that of the 110 kg/m^3^ and 200 kg/m^3^ cores; thus, was much easier to deform and fail, which decreased the energy absorption. In addition, it is clear that both a downward and an upward trend were observed in the graphs of the EA for the sandwich beams with cores of 10 mm and 20 mm and face sheets with thicknesses of 0.5 mm and 1.0 mm, respectively, as shown in Figure 11c–f.

### 3.3. Failure Modes and Deformation Mechanisms

#### 3.3.1. Quasi-Static Load Case: Failure Modes and Deformation Mechanisms

**Figure 12:**
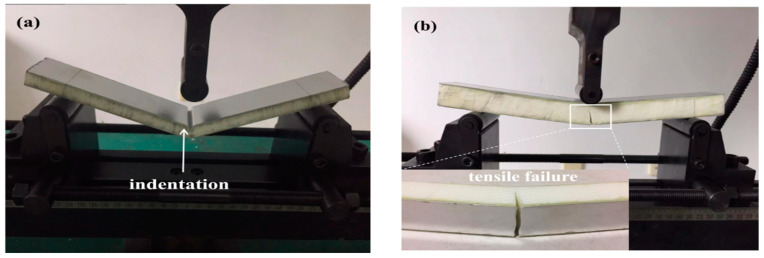
Typical failure modes of the three-point bending tests: (**a**) indentation failure mode of A-0.5/10/0.5; (**b**) typical tensile failure mode of C-0.5/10/0.5.

Figure 12 illustrates the failure modes of the sandwich beams under a three-point bending load. Nearly all of the sandwich beams failed in the local indentation failure mode except for two specimens: C1-0.5/10/0.5 and C2-0.5/20/0.5, with cores of 200 kg/m^3^ in density, which failed in the bottom face sheet tensile failure modes. The top face sheet was made of aluminum, and it was either 0.5 mm or 1 mm in thickness. Thus, the face sheet had a lower degree of stiffness. When the press-head of the three-point bending test acted on the sandwich beam, the load was transferred from the top face sheet to the core due to the low stiffness of the top face sheet. Then, a local compressive load acted on the core directly. If the core had enough strength, the core and the bottom face sheet would deform in a bending deformation. Otherwise, a local indentation failure would occur at the position of the press-head on the core. Therefore, for the cores that were 52 kg/m^3^ and 110 kg/m^3^ in density, the corresponding failure mode was that of a local indentation failure mode.

However, for the core that was 200 kg/m^3^ in density, the core had enough strength to sustain the local press load. When the core and bottom face sheet started to bend together, the bottom face sheet was in the tensile mode. For C1-0.5/10/0.5 and C2-0.5/20/0.5, the bottom face sheet of 0.5 mm thickness failed much more easily in tensile mode. Subsequently, the cores of specimens C1 and C2 also failed in a tensile mode, following the tensile failure of the bottom face sheet.

For specimen C3-1.0/10/1.0 and C4-1.0/20/1.0, a bottom face sheet of 1.0 mm was able to bear adequate tensile stress whereas the local press load made the core failure in an indentation mode.

A modified Gibson’s model was used to analyze the experimental results, and a failure-mode map can be drawn using the equations presented in Ref. [3]. Additionally, the corresponding critical failure load and maximum deflection of the prediction failure modes can be found in Ref. [3], and they are calculated and listed in Table 2.

**Figure 13:**
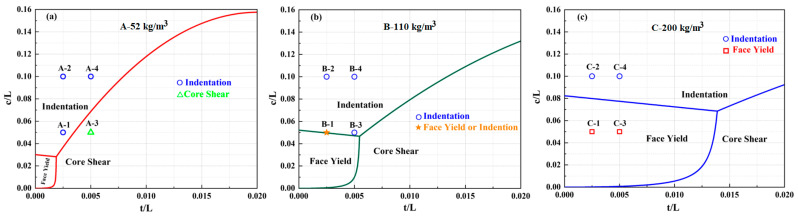
Failure mode maps of the sandwich beams with core densities of: (**a**) 52 kg/m^3^; (**b**) 110 kg/m^3^; (**c**) 200 kg/m^3^.

Figure 13 presents a comparison of the predicted failure-mode maps with the quasi-static results. The region of the face yield is expanded, while the region of the indentation and core shear shrink when the core density is increased, as depicted in Figure 13a–c. Specimen A3-1.0/10/1.0 collapsed in an indentation mode, not a core shear mode, as predicted theoretically in Figure 13a. However, all specimens with a core of 110 kg/m^3^ in density are located in the region of the indentation mode, which indicates that the theoretical prediction is a good agreement with the quasi-static experimental results, as shown in Figure 13b and listed in Table 2. While specimens C2-0.5/20/0.5 and C3-1.0/10/1.0 were deformed in the test and experienced tensile failure, the indentation modes were not deformed in an indentation or face yield mode as predicted theoretically in Figure 13c. This indicates that our current model failure map inaccurately predicted the failure modes experienced by the sandwich beam. The theoretical model may have disregarded the influence of the face sheet and core elasticity, strain hardening, and inertia [30], which will be the focus of future work. Therefore, it can be concluded that the predicted failure modes agreed with the experimental results for all of the specimens except A-3, C-2, and C-3.

#### 3.3.2. Impact Load Case: Failure Mode and Deformation Mechanism

**Figure 14:**
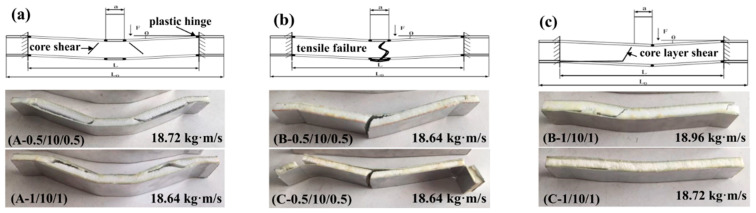
Failure modes and deformation mechanisms of the thin-core sandwich beams with different thicknesses of face sheets and different densities. (**a**) 52 kg/m^3^; (**b**) 110 kg/m^3^; (**c**) 200 kg/m^3^.

**Figure 15:**
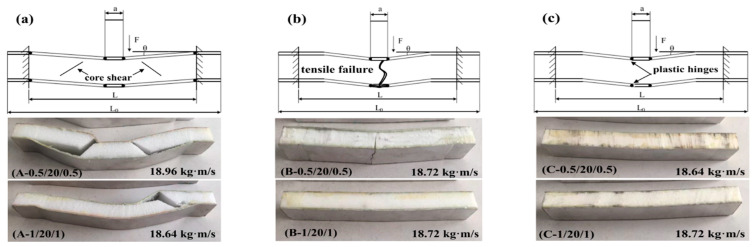
Failure modes and deformation mechanisms of the thick-core sandwich beams with different thickness of face sheets and different densities. (**a**) 52 kg/m^3^; (**b**) 110 kg/m^3^; (**c**) 200 kg/m^3^.

Figure 14 and Figure 15 illustrate with schematic graphs the failure mechanisms of the sandwich beams with cores of 10 mm and 20 mm in thickness, respectively. Under impact loading, the failure modes of the sandwich beams included shear failure mode and tensile failure mode, which was different from the indentation failure mode under the quasi-static load case. Additionally, the load transferring mechanism of the impact load case was different from that of the quasi-static load case.

When the impact loading acted on a sandwich beam, a plastic hinge was formed on the upper face sheet, as shown in Figure 14 and Figure 15. Additionally, the load was transferred to the core and bottom face sheet due to the low level of stiffness of the face sheet that was 0.5/1 mm thick. Subsequently, the thickness of the PMI core, the density of the PMI core, and the thickness of the bottom face sheet played a critical role in the failure mode of the sandwich beam, which took precedence over the sandwich beam’s stiffness. A low level of stiffness would result in a PMI core shear failure mode, and a bottom face sheet tension failure mode was caused by a greater level of stiffness. It was clear that the core sustained the shear stress, and the upper and bottom face sheets suffered from compressive and tensile stresses, respectively. Thus, the failure mode depended on the stiffness of the sandwich beam, which resulted in the different failure modes of the core and the bottom face sheet.

For the core of 52 kg/m^3^, the strength was much lesser than that of the 110 kg/m^3^ and the 200 kg/m^3^ cores, and the corresponding stiffness was also lesser compared to that of the bottom face sheets of 0.5 mm and 1.0 mm under the tension stress; the PMI core of 52 kg/m^3^ failed in shear failure mode first. This finding agreed well with the experimental results, as shown in Figure 14a and Figure 15a.

For the cores of 110 Kg/m^3^ and 200 Kg/m^3^, there were two cases: (1) The strength of the core was enough to bear the shear stress, in which case, the bottom face sheet failed in tensile failure mode when the tensile stress exceeded the strength of the bottom face sheet, for instance, in the case of the bottom face sheet of 0.5 mm in thickness, as shown in Figure 14b and Figure 15b; (2) The bottom face sheet was able to bear the tensile stress due to a thick core of 20 mm, but in this case, the shear stress in the PMI core exceeded the strength of the PMI, which made the PMI core fail in shear failure mode, such as in the case of the bottom face sheet of 0.5 mm in thickness, as shown in Figure 14c and Figure 15c.

## 4. Conclusions

The deformation and failure mechanisms of the sandwich beams with PMI foam cores were investigated under different load cases. For the quasi-static load case, the peak force increased with increases in the density of the core and in the thickness of the face sheet. Moreover, most of the sandwich beams failed in an indentation failure mode, except for C-0.5/10/0.5 and C-0.5/20/0.5, which failed in a bottom face sheet tensile failure mode. The reason was that the upper face sheet did not diffuse the load due to its low level of stiffness, and the concentrated load was transferred to the PMI core, which resulted in an indentation failure mode. For the impact load case, both the peak value and duration time of the impact force were also influenced by the density of the core, which increased with an increase in the density of the core. The stiffness of the sandwich beam was improved by a PMI core with an increased density, which resulted in a bending deformation for the sandwich beam. Additionally, the load was sustained by the bottom face sheet in a tension status. Thus, the failure mode of the sandwich beam varied from shear failure to the tensile failure, which was governed by the component that failed first: the core or the bottom face sheet.

Based on the above-discussed results, a high-performance sandwich beam with a PMI foam core could be designed by modulating the bending-resistance of the sandwich beam, which includes both the thickness and strength of the face sheet and the PMI foam core. Until now, most studies have focused on sandwich beams with symmetrical structures or uniform cores. In future research, asymmetric sandwich beams or graded PMI foam cores could be investigated to improve the performance of sandwich beams.

## Figures and Tables

**Table 1:** Sizes of face sheet and foam core, along with the weight of the tested specimen.

Specimen Code	Sandwich Structure (mm)	Face Sheet Size (mm)	Foam Core Size (mm)	Weight of the Specimen (kg)
A-1	0.5/10/0.5	260 × 40 × 0.5	260 × 40 × 10	0.046
A-2	0.5/20/0.5	260 × 40 × 0.5	260 × 40 × 20	0.052
A-3	1.0/10/1.0	260 × 40 × 1.0	260 × 40 × 10	0.057
A-4	1.0/20/1.0	260 × 40 × 1.0	260 × 40 × 20	0.081
B-1	0.5/10/0.5	260 × 40 × 0.5	260 × 40 × 10	0.054
B-2	0.5/20/0.5	260 × 40 × 0.5	260 × 40 × 20	0.066
B-3	1.0/10/1.0	260 × 40 × 1.0	260 × 40 × 10	0.081
B-4	1.0/20/1.0	260 × 40 × 1.0	260 × 40 × 20	0.090
C-1	0.5/10/0.5	260 × 40 × 0.5	260 × 40 × 10	0.061
C-2	0.5/20/0.5	260 × 40 × 0.5	260 × 40 × 20	0.078
C-3	1.0/10/1.0	260 × 40 × 1.0	260 × 40 × 10	0.083
C-4	1.0/20/1.0	260 × 40 × 1.0	260 × 40 × 20	0.094

**Table 2:** Quasi-static experimental results.

No.	Specimen Code	Sandwich Structure	Peak Load (N)	Deflection (mm)	Theoretical Value of Peak Load (N)	Theoretical Value of Deflection (mm)	Failure Mode
1	A-1	0.5/10/0.5	388.50	2.59	447.21	2.82	Indentation
2	A-2	0.5/20/0.5	603.66	3.22	447.21	2.21	Indentation
3	A-3	1.0/10/1.0	573.26	3.03	894.43	4.19	Indentation
4	A-4	1.0/20/1.0	891.29	4.73	894.43	4.07	Indentation
5	B-1	0.5/10/0.5	609.87	6.82	774.59	6.20	Indentation
6	B-2	0.5/20/0.5	1217.52	2.75	774.59	2.21	Indentation
7	B-3	1.0/10/1.0	1016.82	3.03	1549.19	3.16	Indentation
8	B-4	1.0/20/1.0	1579.04	3.74	1549.19	3.0	Indentation
9	C-1	0.5/10/0.5	889.19	12.17	834.75	12.94	Tensile failure
10	C-2	0.5/20/0.5	1912.09	10.73	2031.25	10.27	Tensile failure
11	C-3	1.0/10/1.0	1300.20	6.67	2449.50	6.10	Indentation
12	C-4	1.0/20/1.0	2916.96	5.46	2449.50	5.13	Indentation

## Data Availability

The raw data needed to reproduce these findings cannot be shared at this time, as the data will be used in ongoing research.
